# Lactate and BDNF: Key Mediators of Exercise Induced Neuroplasticity?

**DOI:** 10.3390/jcm9041136

**Published:** 2020-04-15

**Authors:** Patrick Müller, Yves Duderstadt, Volkmar Lessmann, Notger G. Müller

**Affiliations:** 1 Research Group Neuroprotection, German Center for Neurodegenerative Diseases (DZNE), Leipziger Str. 44, 39120 Magdeburg, Germany; yves.duderstadt@dzne.de (Y.D.); notger.mueller@dzne.de (N.G.M.); 2 Medical Faculty, Department of Neurology, Otto-von-Guericke University, Leipziger Str. 44, 39120 Magdeburg, Germany; 3 Medical Faculty, Institute of Physiology, Otto-von-Guericke University, Leipziger Str. 44, 39120 Magdeburg, Germany; volkmar.lessmann@med.ovgu.de; 4 Center for Behavioral Brain Sciences (CBBS), Leipziger Str. 44, 39120 Magdeburg, Germany

**Keywords:** physical exercise, BDNF, lactate, neuroplasticity

## Abstract

Accumulating evidence from animal and human studies supports the notion that physical exercise can enhance neuroplasticity and thus reduce the risk of several neurodegenerative diseases (e.g., dementia). However, the underlying neurobiological mechanisms of exercise induced neuroplasticity are still largely unknown. One potential mediator of exercise effects is the neurotrophin BDNF, which enhances neuroplasticity via different pathways (e.g., synaptogenesis, neurogenesis, long-term potentiation). Current research has shown that (i) increased peripheral lactate levels (following high intensity exercise) are associated with increased peripheral BDNF levels, (ii) lactate infusion at rest can increase peripheral and central BDNF levels and (iii) lactate plays a very complex role in the brain’s metabolism. In this review, we summarize the role and relationship of lactate and BDNF in exercise induced neuroplasticity.

## 1. Introduction

Physical activity and/or exercise (Table 1) is a low-cost intervention in primary and secondary prevention for numerous chronical diseases (e.g., diabetes, cancer, cardiovascular diseases, sarcopenia) [1]. Furthermore, physical activity has several positive effects on brain health, can stimulate neuroplasticity and reduce the risk of neurodegenerative diseases (e.g., dementia) [2,3,4,5,6]. Especially in the context of an unavailable causal pharmacological treatment for dementia, research is currently focusing on modifiable risk and lifestyle factors that can delay the outbreak of the disease or ameliorate associated memory deficits [7,8]. In western societies physical inactivity is one central modifiable risk factor for dementia [9] and thus target for interventions. In the last decades, the impact of exercise on brain health has become a central research project in neuroscience and several lines of evidence suggest that BDNF is one of the essential mediating factors of exercise induced neuroplasticity. However, the underlying neurobiological and molecular mechanisms of exercise induced neuroplasticity are still largely unknown [10]. Interestingly, recent results from animal and human research suggest that lactate might link physical exercise to BDNF-dependent neurobiological pathways [11,12,13,14].

## 2. BDNF and Neuroplasticity

The protein brain-derived neurotrophic factor [BDNF] belongs to the family of neurotrophins (NT), which in mammals also comprise nerve growth factor [NGF], neurotrophin 3 [NT-3] and NT-4/5. Neurotrophins crucially regulate important neurobiological processes such as neurogenesis, synaptogenesis, growth of dendritic spines, long-term potentiation [LTP] and efficacy of protein synthesis [19,20,21,22,23,24]. BDNF is synthesized in the endoplasmic reticulum (ER) as a pre-cursor protein (calculated molecular weight 28 kDa). After initial cleavage of the pre-sequence, the resulting proBDNF (calculated molecular weight 26 kDa) undergoes further processing (e.g., glycosylation) in the ER and in the Golgi apparatus (reviewed in [25]), including partial endoproteolytic cleavage of the pro-form which eventually yields mature BDNF (mBDNF; commonly termed as BDNF; calculated molecular weight 13.5 kDa). Accordingly, proBDNF and mBDNF are co-stored in vesicles that bud-off from the trans-Golgi network and are transported to the cell membrane where they undergo exocytosis, thus leading to secretion of proBDNF, mBDNF and smaller cleavage products into the extracellular space. These vesicles belong either to the constitutive or the regulated secretory pathway (see below). However, the exact composition of BDNF species in secreted vesicles is yet unknown. Released proBDNF can be converted in the extracellular space by endoproteases to mBDNF [25]. While mBDNF binds selectively to the tyrosin-related kinase receptor B [TrkB], proBDNF binds specifically to the so-called p75 neurotrophin receptor [p75^NTR^] of target cell membranes. Because of the antagonistic effects of proBDNF/p75 signaling (mediating apoptosis, shrinkage of dendritic spines, and long-term depression [LTD] compared to mBDNF/TrkB signaling pathways, (supporting neuronal survival, synaptogenesis, growth of dendritic spines, and LTP) [26] the proportion of released proBDNF and mBDNF critically determines the direction of neuroplasticity that can be elicited in target neurons (for further information on BDNF synthesis, processing, and expression see [27,28]). Activation of TrkB triggers intracellular signal cascades such as mitogen-activated protein kinase (MAPK), phospholipase C-γ (PLCγ) or phosphatidylinositol-3-kinase (PI_3_K) pathways [29]. Additionally, TrkB activation can also increase the expression of the peroxisome proliferator-activated receptor γ co-activator α (PGC1α) which in turn increases BDNF expression in neurons via the PGC1α/FNDC5/BDNF pathway [30,31].

As mentioned above, BDNF release can take place via two classes of vesicles. The Golgi-derived vesicles of the constitutive pathway of secretion undergo exocytosis by default when they reach the plasma membrane. In contrast, activity-dependent release of BDNF (e.g., driven by repetitive firing of action potentials) from Golgi-derived vesicles of the regulated pathway of secretion requires sustained intracellular Ca^2+^ elevations [32]. In the brain, most of the released BDNF is secreted via the regulated pathway [23,25]. Importantly, under physiological conditions, intracellular BDNF protein levels are extraordinarily low in many brain regions. In rodents, highest levels are observed in the hippocampal CA3 and dentate gyrus area, the amygdala, and selected regions of the cerebral cortex, including the visual and somatosensory cortex [27,28,33,34,35]. The low expression level and the largely activity-dependent and locally restricted release give BDNF a strong command in fine-tuning cellular functions with high spatial selectivity [23,25].

In the central nervous system (CNS) only excitatory glutamatergic (but not inhibitory GABAergic) neurons have the capacity to synthesize BDNF. However, GABAergic neurons are dependent on extracellular BDNF for survival and synaptogenesis [24,36]. In addition, microglial cells, T and B lymphocytes, monocytes and skeletal muscle cells can synthesize and release BDNF [27,37,38]. Thrombocytes cannot synthesize, but seem to take up and store BDNF via receptor-mediated endocytosis [39,40,41]. BDNF can pass the blood-brain barrier [42] and about 75% of the peripheral BDNF plasma level originates from the brain [43,44]. This is the reason why blood plasma (and serum) BDNF levels are considered to serve as a proxy for released BDNF in the brain and can thus be used to investigate the effect of lifestyle interventions (e.g., physical exercise, caloric-restricted diet) on BDNF-mediated neuroplasticity in the brain.

BDNF mediates neuroplasticity via different mechanisms and on distinct time scales [27,29]. Thus, BDNF/TrkB signaling induces within seconds to minutes LTP at glutamatergic synaptic connections [45,46] and the growth of new synaptic spines [47,48], thereby enabling the initial formation of new memory traces at existing synapses. On the time scale of hours, BDNF contributes to consolidation of protein synthesis dependent long lasting LTP and memory formation, and on even longer time scales shapes memory engrams by incorporating newborn neurons into neuronal circuits by promoting neuro- and synaptogenesis. In these ways, BDNF signaling crucially contributes to cellular mechanisms of neuronal plasticity that drive formation, consolidation and retrieval of memory.

In humans, BDNF has been associated with psychiatric [49] (e.g., schizophrenia, major depressive disorder, anxiety disorders) and neurological diseases (e.g., dementia [50,51], Huntington‘s disease [52]). Reduced BDNF levels have been reported in the hippocampus [53] and in the blood [54,55,56] in patients with mild cognitive impairment (MCI) or Alzheimer‘s disease (AD). In contrast, some studies have found no association of BDNF and AD [57]. Based on a community-based, prospective cohort study with 2131 dementia-free participants Weinstein et al., proposed that higher serum BDNF levels may protect against dementia [58]. However, these cross-sectional data do not allow a causal interpretation. A common (observed in approx. 30% of humans) single nucleotide polymorphism (SNP) in the BDNF gene that leads to a substitution of valine to methionine in the pro-region has been associated with reduced activity-dependent BDNF secretion [59]. Nonetheless, a higher incidence of the above mentioned neurological and neuropsychiatric disorders in carriers of the SNP has thus far remained elusive. This might at least in part result from the physical exercise-dependent regulation of BDNF protein expression and release (see below) that might overcome the decreased basal secretion of Val66Met BDNF [59].

## 3. Physical Exercise and BDNF

Physical exercise can improve cognition (e.g., executive functions, spatial memory, learning) [60,61,62,63,64] and induce structural and functional brain plasticity [65,66,67,68,69,70,71,72,73,74,75,76]. Epidemiological, observational and intervention studies indicate that exercising can reduce the risk of neurodegenerative diseases (e.g., dementia [2,4,77], multiple sclerosis [78], Parkinson’s disease [79,80]). Proposed mechanisms of exercise induced neuroplasticity are increased expression, secretion, and downstream signaling of neurotrophic factors (e.g., BDNF, VEGF, IGF-1), reduced inflammation [81,82], reduced stress levels [83] and improved cardiovascular (e.g., reduced arterial stiffness, reduced blood pressure) [84,85] and metabolic (e.g., insulin sensitivity) [86,87] parameters. Numerous studies have shown that exercise induced neuroplasticity is associated with BDNF [88,89]. Indeed, at least in rodents, pharmacological blocking of BDNF signaling in the hippocampus attenuates the neuroplastic effects of physical exercise [90].

Neeper et al., were the first to report a positive correlation between physical activity and BDNF mRNA levels in rodents [91]. In animal models, short and long term periods of exercise increase BDNF gene and protein expression in the hippocampus [92,93] and other brain structures (e.g., amygdala [94], cerebellum [92], perirhinal cortex [95]). However, the effect of exercise on BDNF expression is smaller in aged animals compared to young ones [96]. Furthermore, Choi et al., reported that exercise induced adult hippocampal neurogenesis is associated with improved cognition, reduced ß-amyloid in the brain and increased levels of BDNF in an AD mouse model [97]. Similar to rodents, physical activity increases peripheral BDNF levels in healthy humans [98,99], and numerous studies have shown a positive impact of acute exercise on BDNF plasma [43,100,101] and/or serum levels [102,103]. Similarly, also chronic exercise increases BDNF plasma [104,105] and/or serum levels [106]. Several reviews and meta-analyses have investigated the effects of physical exercise on BDNF blood levels [98,99,107,108,109,110,111]. Overall, they strongly suggest that acute [43,112,113] and chronic [99,114,115] exercise can increase peripheral BDNF levels in humans. Regarding acute exercise the duration and intensity of exercise correlate with larger increases in BDNF [112,113]. Additionally, effect sizes in women seem to be significantly smaller after acute exercise [116]. In contrast, no gender effect was reported following chronic exercise [115]. A recent meta-analysis reported that aerobic exercise alone does not increase BDNF in older adults while resistance exercise and combined aerobic/resistance exercise increases peripheral BDNF levels [117].

## 4. Physical Exercise, Lactate and BDNF

Several trials have used blood lactate for the monitoring of exercise intensity. These studies indicate that higher lactate concentrations are associated with increased BDNF plasma and/or serum levels [102,118,119]. Furthermore, current evidence indicates that high intensity interval training evokes larger BDNF levels compared to moderate and/or intensive continuous exercise [110,112].

For long, lactate was simply considered a waste product of the anaerobic metabolism. Nowadays, however, it is clear that lactate is an important signaling molecule that is involved in several metabolic processes [14,120]. Energy supply for exercise is based mainly on three pathways: (i) ATP-Creatinkinase, (ii) glycolysis and (iii) oxidative phosphorylation [121]. Lactate is produced by glucose oxidation when oxygen uptake is low, and it can buffer acidosis. Accumulated lactate can be transported to the liver (where lactate is synthesized to glucose through gluconeogenesis) or can be directly used as a fuel by muscles, heart and brain [122]. During acute exercise lactate accumulates depending on the intensity and the duration of the exercise. The lactate threshold (also called anaerobic threshold) is defined as the highest level of physical activity that can be achieved without lactate accumulation and is a predictor of an individual’s fitness level. Physical exercise can improve the fitness level and can increase the lactate threshold [123].

Lactate can cross the blood-brain barrier (BBB) [120] reaching neurons via monocarboxylate transporters (MCT) [124,125,126]. MCT 2 is the major transporter in neurons [127] while MCT 4 is only expressed in astrocytes [128]. Astrocytes have complex interactions with neurons. They are involved in the control of cell volumes, energy metabolism and ionic homeostasis [129]. Astrocytes show a glucose gradient with high glucose concentrations close to the BBB and low glucose concentrations close to neurons. This gradient allows a rapid glucose transfer to neurons. Furthermore, astrocytes can store glycogen and support the neuronal energy metabolism [126,130,131].

Additionally, Pellerin et al., proposed an astrocyte-neuronal lactate transport during excitatory neurotransmission [132]. Here lactate is transported from astrocytes to neurons through MCTs where lactate is converted to pyruvate and enters the tricarboxylic acid cycle. Lactate in neurons can origin from astrocyte metabolism or from peripheral muscle activity. Moreover, neurons in vitro prefer lactate instead of glucose [133]. Current research indicates that lactate transport from astrocytes to neurons plays a crucial role for memory formation [134,135,136] and could be a link between exercise and neuroplasticity [120]. Pharmacological inhibition of MCT 2 irreversibly impairs long-term memory [136]. Van de Hall et al. [125] have shown that lactate uptake in the brain increases from 8% at rest up to 20% during exercise. Additionally, Kemppainen et al. [137] reported a higher lactate metabolism in trained healthy adults compared to controls. In rodents a single bout of exercise can induce up-regulation of MCTs [138]. Proia et al., hypothesized that exercise can increase levels of BDNF and of other growth factors such as insulin-like growth factor 1 (IGF-1) [120] and vascular endothelial growth factor (VEGF) [139,140,141].

**Figure 1:**
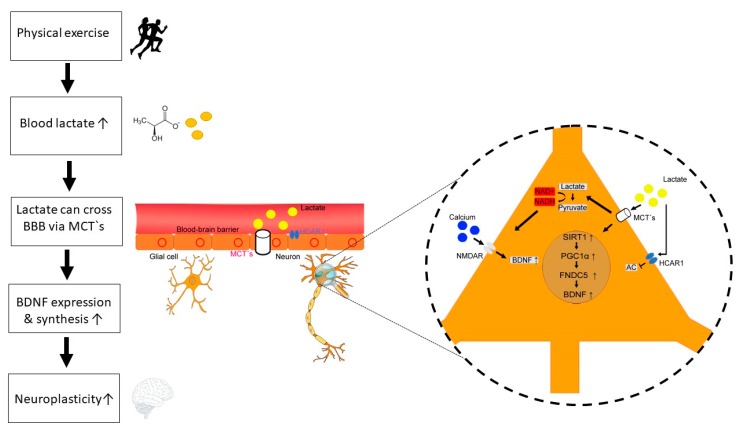
Potential mechanisms of lactate-BDNF interaction following physical exercise. Physical exercise is associated with intensity-dependent increase of lactate levels. Lactate can cross the blood-brain barrier (BBB) via different monocarboxylate transporters (MCT‘s). Furthermore, lactate binding to the hydroxycarboxylic acid receptor (HCAR1) at the BBB can induce angiogenesis. In neurons, lactate exerts several neurotrophic and metabolic effects through transmembrane transport via MCT’s and direct binding to HCAR1. Firstly, lactate binding to HCAR1 on neurons inhibits the adenylate cyclase (AC) and thus decreases cAMP, resulting reduced BDNF expression and regulatory function in the control of blood flow, and synaptic functions. Secondly, lactate can induce the PGC1α/FNDC5/BDNF pathway through SIRT1 activation. Thirdly, lactate increases intracellular NADH, resulting in enhanced calcium levels and BDNF gene expression. Released BDNF can then enhance neuroplasticity via different neurobiological mechanisms (e.g., neurogenesis, synaptogenesis, growth of dendritic spines, long-term potentiation [LTP]).

However, the interaction between lactate and BDNF levels is not yet well resolved. Potential mechanisms that link both molecules could be (i) a lactate regulated increased NMDA-receptor activation and as a consequence increased intracellular calcium levels, (ii) a signaling cascade initiated by lactate binding to different G-protein coupled receptors (GPCR), and (iii) through silent information regulator 1 (SIRT1) activation of the PGC1α/FNDC5/BDNF pathway (Figure 1).

(i) Yang et al., reported that lactate promotes plasticity related gene expression by potentiating NMDA glutamate receptor activity in neurons [142]. Furthermore, lactate increases intracellular NADH and calcium levels. This could be a central mechanism for neuroplasticity induced by lactate from astrocytes. Additionally, increased intracellular calcium following lactate induced enhanced NMDA receptor activity could be a link between exercise and BDNF expression. [25].
(ii) Lactate can bind to GPCR81 (also known as hydroxycarboxylic acid receptor [HCAR1]) on neurons [143] and at the BBB [144]. Lauritzen et al., have shown, that HCAR1 at the BBB is essential for mediating exercise effects on angiogenesis in a mouse model [144]. Furthermore, lactate binding to HCAR1 on neurons inhibits the adenylate cyclase and thus decreases cAMP, resulting in reduced neuronal activity and gene regulation [14,143,145]. Here, lactate could have a metabolic and regulatory function in the control of blood flow and synaptic function [146]. Furthermore, lactate can influence prostaglandin E2 uptake and thus influence vasodilation [147]. The potential negative modulation of BDNF production by lactate through HCAR1 should be examined more closely in the future.
(iii) Lactate can induce the PGC1α/FNDC5/BDNF pathway through SIRT1activation [11]. El Hayek et al., have shown, that voluntary exercise promotes hippocampal BDNF expression and improves memory and learning in a lactate-dependent manner in rodents [11]. Furthermore, they have shown that intraperitoneal lactate infusion in mice induces SIRT1 activity and thus enhances the PGC1α/FNDC5/BDNF pathway which results in improved spatial learning and memory retention.

Schiffer et al. [12] investigated whether lactate infusion at rest can elevate BDNF blood concentration in young adults. The lactate clamp method is a well-established method to examine (neuro-) physiological effects of lactate without stimulating physical exercise. After infusion of a 4 molar sodium-lactate solution, BDNF serum and lactate levels increase significantly and returned to baseline values at follow-up [12]. Potential mechanisms of BDNF serum increase after lactate infusion could be (i) a lactate driven BDNF expression or (ii) a release of BDNF from platelets (in context of blood gases disturbance).

## 5. Conclusions

Physical exercise induces numerous metabolic adaptations. However, most studies have investigated the effects of acute and/or chronic exercise on brain plasticity and underlying mechanism with a limited test battery only (e.g., neuropsychological tests, magnetic resonance imaging [MRI] and BDNF). To better understand the neurobiological mechanism induced by exercise, more extensive assessments are necessary. For instance, research studies in rodents and/or humans have shown that exercise can enhance cathepsin B [148], FNDC5/Irisin [31], lactate and BDNF, while the mechanisms interconnecting these observations are yet not understood. Future studies are urgently needed that combine animal and human exercise research with extensive test batteries to foster our understanding on how exercise can induce neuroplasticity. Identifying the underlying molecular mechanisms will help to design (i) more tailored exercise interventions [149] and (ii) pharmacological agents that can mimic the effects of physical exercise [150].

## Figures and Tables

**Table 1:** Physical activity and physical exercise.

Physical Activity and Physical Exercise-Where is the Difference?
“Physical activity”’ is defined as any muscle-induced bodily movement which increases energy expenditure above ~1.0/1.5 metabolic equivalent of task (MET, 1 MET = 1 kcal (4184 kJ) × kg^−1^ × h^−1^) whereby “physical exercise” is a specific, planned and structured form of physical activities [15,16]. Additionally, physical exercise can be divided into acute physical exercise (single bout) and chronic physical exercises (repeated single bouts). Current guidelines recommend a minimum of 150 min moderate-intensity or 75 min vigorous-intensity aerobic activity and strength training per week [17,18].

## Abbreviations

AD	Alzheimer’s disease
BBB	blood-brain barrier
BDNF	brain-derived neurotrophic factor
CNS	central nervous system
HCAR1	hydroxycarboxylic acid receptor 1
ER	endoplasmic reticulum
FNDC5	fibronectin type III domain-containing 5
LTD	long-term depression
LTP	long-term potentiation
GPCR	G protein coupled receptors
MAPK	mitogen-activated protein kinase
mBDNF	mature brain-derived neurotrophic factor
MCI	mild cognitive impairment
MCT	monocarboxylate transporter
MET	metabolic equivalent of task
NGF	nerve growth factor
NMDA	*N*-Methyl-d-aspartate
NT-3	neurotrophin-3
NT-4/5	neurotrophin-4/5
PGC1α	peroxisome proliferator-activated receptor γ co-activator α
PI3K	phosphatidylinositol-3-kinase
PLCγ	phospholipase C-γ
SIRT1	silent information regulator 1
SNP	single nucleotide polymorphism
TrkB	tyrosine-kinase receptor B
